# CD7-Positive Diffuse Large B-Cell Lymphoma Presenting as an Intranasal Tumor

**DOI:** 10.1155/2020/1514729

**Published:** 2020-04-12

**Authors:** Kazuaki Teshima, Masaaki Kume, Yasushi Kawaharada, Takashi Saito, Ko Abe, Sho Ikeda, Hideaki Ohyagi, Megumi Zuguchi, Masashi Zuguchi, Yosuke Kubota, Yoshitaka Enomoto, Masahito Miura, Satsuki Takahashi, Masahiro Saito, Ken Saito, Naoto Takahashi

**Affiliations:** ^1^Department of Hematology, Hiraka General Hospital, Yokote, Japan; ^2^Department of Hematology, Nephrology, and Rheumatology, Akita University Graduate School of Medicine, Akita, Japan; ^3^Department of Surgery, Hiraka General Hospital, Yokote, Japan; ^4^Department of Otolaryngology, Hiraka General Hospital, Yokote, Japan; ^5^Department of Gastroenterology, Hiraka General Hospital, Yokote, Japan; ^6^Department of Diagnostic Pathology, Hiraka General Hospital, Yokote, Japan

## Abstract

We report a case of a 74-year-old man with a cluster of differentiation (CD) 7-positive diffuse large B-cell lymphoma (DLBCL) in the right nasal cavity. Flow cytometry analyses showed CD7 and CD20 positivity in tumor cells. The patient received 6 cycles of R-CHOP plus local radiation therapy because positron emission tomography-computed tomography after R-CHOP revealed an intranasal lesion. The patient achieved complete remission (CR) after radiation therapy. The frequency of CD7-positive DLBCL is rare, and only 11 cases with follow-up of clinical course have been reported thus far. CR or partial response was noted in 8 of 11 cases after receiving rituximab combined with chemotherapy. In total, 9 of 12 cases involved the development of extranodal lesions, which occurred as an intranasal tumor in 3 cases. It is important to examine the clinical features by accumulation of further cases.

## 1. Introduction

Aberrant expression of T-cell markers (CD2, CD3, CD4, CD5, CD7, and CD8) is occasionally observed in diffuse large B-cell lymphoma (DLBCL) [1]. In particular, expression of CD5 is noted in approximately 10% DLBCL cases, and it is related to a poor prognosis [2]. However, the clinical significance of the expression of other T-cell markers, including CD7, is unclear. Here, we report a rare case of CD7-positive DLBCL presenting as an intranasal tumor.

## 2. Case Presentation

**Figure 1:**
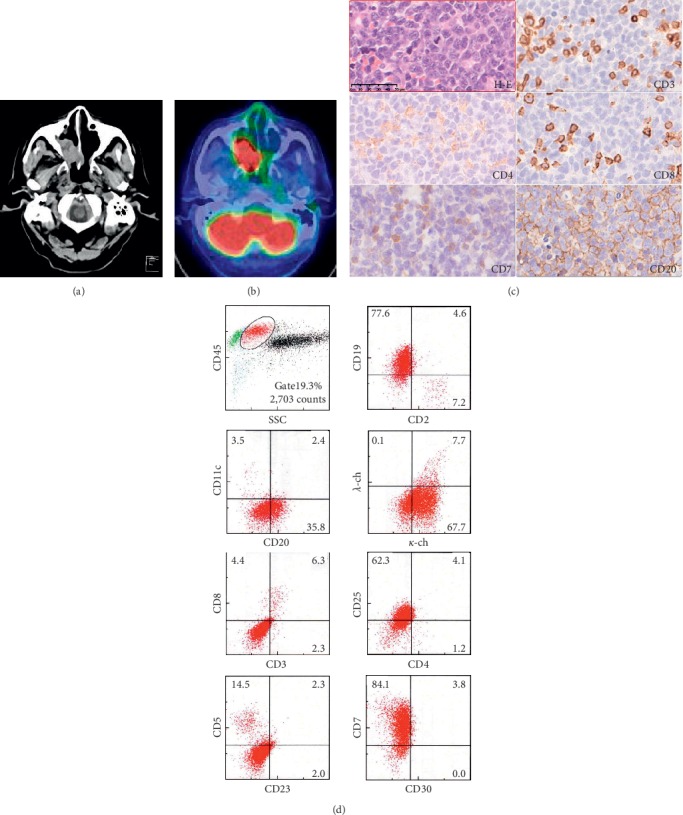
Imaging findings: (a) brain computed tomography scan at diagnosis; (b) positron emission tomography-computed tomography findings at diagnosis; (c) pathological findings at diagnosis (hematoxylin and eosin staining) and immunostaining with anti-CD3, anti-CD4, anti-CD8, anti-CD7, and anti-CD20; (d) flow cytometry analysis at diagnosis.

A 74-year-old male patient with a history of left lung inferior lobe resection due to localized lung cancer who complained of rhinostenosis was diagnosed with a tumor in the right nasal cavity (Figure 1(a)). Positron emission tomography-computed tomography (PET-CT) showed intense focal uptake of SUVmax 18.3 in the right nasal cavity (Figure 1(b)). The tumor biopsy showed diffuse proliferation of large, atypical lymphoid cells. Flow cytometry (FCM) analyses showed clear CD7 and CD19 positivity in the tumor cells, while CD20 was expressed at weak intensity (Figure 1(d)). These data were analyzed by FACSCanto II (BD Biosciences) using the antibody of CD7 (3A1-RD1; Beckman Coulter). These data were obtained from the SRL Laboratory (Hachioji city, Tokyo, Japan). Immunohistochemistry (IHC) results were positive for L26 (CD20), bcl-2, bcl-6, and MUM1 and negative for CD3, CD4, CD8, CD7 (LP15; Novocastra), CD10, and EBER (Figure 1(c)). Fluorescent in situ hybridization (FISH) did not reveal IgH/c-MYC rearrangement. Southern blotting revealed immunoglobulin heavy-chain gene rearrangement, however not T-cell receptor (TCR) gene rearrangement. Bone marrow biopsy was negative for the involvement of atypical large cells. The patient was diagnosed with stage IA DLBCL. The patient received 6 cycles of R-CHOP therapy; however, residual disease was found on the PET-CT. Therefore, the patient received local radiation therapy for the residual disease. He achieved complete remission after radiation therapy. At 18 months after chemoradiation therapy, there was no evidence of recurrence.

## 3. Discussion

CD7 is expressed on the surface of most thymocytes and T-cells preceding TCR-*β*-chain gene rearrangement and is the earliest differentiation antigen found during T-cell development [3]. Aberrant T-cell marker expression is occasionally observed in B-cell lymphoma [4]. Inaba et al. reported that CD7 was expressed in 5 (5%) of 101 DLBCL patients [5].  Table 1 describes the clinical characteristics of 12 cases, including the present case, of CD7-positive DLBCL reported thus far. Two cases were detected by FCM, 1 case was detected by IHC, and 9 cases were detected with both. In the present case, FCM showed CD7 positivity and IHC showed CD7 negativity. There are several possible causes for CD7-negative immunostaining results. One reason is that the antigenicity of CD7 may decline in the process of formalin fixation. Another reason is that epitope of lymphoma may be different from that of normal cells. FCM shows CD7 expressions despite no expression of CD3. Clonal immunoglobulin heavy-chain rearrangement was observed using Southern blotting, which detected no clonal TCR gene rearrangement. This means that clonally restricted B-cells in our DLBCL case express CD7. Suzuki et al. reported that aberrant T-cell marker expression detected by FCM could be confirmed in some, but not in all, cases detected by IHC [6]. They reported that among 6 CD7-positive cases by FCM, only 3 were confirmed to be positive for CD7 by IHC. They considered that it was important to employ FCM because the ability of IHC to detect aberrant T-cell marker expression is restrictive.

Inaba et al. reported that extranodal involvement and a higher international prognostic index are significantly more frequent in B-cell lymphomas positive for T-cell markers than in B-cell lymphomas negative for T-cell markers [5]. Tsuyama et al. reported the clinical examination of 223 DLBCL patients for T-cell marker antigen [7]. According to their report, the CD5-positive DLBCL group had a significantly poorer prognosis than the T-cell marker-negative group. In contrast, there was no significant difference in prognosis between T-cell marker-positive DLBCL other than CD5 and T-cell marker-negative DLBCL. Furthermore, Suzuki et al. reported 150 cases of DLBCL; however, the clinicopathological significance and prognosis of CD7 in DLBCL are still unknown [6].

As shown in Table 1, of the 11 previously reported cases of CD7-positive B-cell lymphoma, 9 cases involved the development of extranodal lesions, including 2 cases of intranasal tumor; 8 patients achieved complete response or partial response after receiving rituximab combined with chemotherapy. Similarly, the patient in the present case presented with an intranasal tumor and achieved complete remission with radiotherapy following R-CHOP therapy. Currently, there is no evidence of recurrence in the patient. According to the literature, CD2 or CD7 expression is associated with extranodal involvement in B-non-Hodgkin's lymphoma at diagnosis [5]. It is important to examine the clinical features by the accumulation of further cases.

## Figures and Tables

**Table 1:** Clinical characteristics of CD7-positive B-cell lymphoma.

Case No.	Age	Sex	Histology	Cell of origin	Main site(s) of involvement	Stage	Extranodal involvement	Treatment	Response	Status	Follow-up (months)	Detection method	References
Case 1	54	F	DLBCL	Non-GC	Inguinal LN	IIA	−	R-CHOP	CR	Alive, CR	NA	FCM, IHC	Sangle et al.
Case 2	66	M	DLBCL	NA	Thoracic cavity	IA	+	CHOP + RT	PR	Died of disease	NA	FCM, IHC	Tomita et al.
Case 3	47	M	Non-Hodgkin lymphoma	NA	Intra-abdominal mass	IVA	+	High dose CHOP	PD	Died of disease	5	FCM	Takahashi et al.
Case 4	64	M	DLBCL	GC	Cecum	IIA	+	Surgery + R-CHOP	CR	Alive, CR	68	IHC	Tsuyama et al.
Case 5	66	F	DLBCL	Non-GC	Axillary LN	IVB	+	R-CHOP + RT	PD	Died of disease, refractory	24	FCM, IHC	Tsuyama et al.
Case 6	62	F	DLBCL	GC	Nasal cavity	IIA	+	R-CHOP	PR	Alive with disease, relapse	34	FCM, IHC	Tsuyama et al.
Case 7	82	M	DLBCL	Non-GC	Nasal cavity	IA	+	R-CHOP	CR	Alive, CR	45	FCM, IHC	Tsuyama et al.
Case 8	64	M	DLBCL	Non-GC	Orbit	IB	+	R-CHOP	PD	Died of disease	2	FCM, IHC	Tsuyama et al.
Case 9	81	M	DLBCL	Non-GC	Soft tissue of leg	IVA	+	R-CHOP	CR	Died of disease, relapse	40	FCM, IHC	Tsuyama et al.
Case 10	55	M	DLBCL	Non-GC	Cervical LN	IVA	+	R-CHOP	CR	Died of disease, relapse	12	FCM, IHC	Tsuyama et al.
Case 11	65	M	DLBCL	Non-GC	Prostate	IVA	+	R-CHOP	CR	Alive, CR	49	FCM, IHC	Tsuyama et al.
Case 12	74	M	DLBCL	Non-GC	Nasal cavity	IA	+	R-CHOP + RT	CR	Alive, CR	18	FCM	Our case

M, male; F, female; DLBCL, diffuse large B-cell lymphoma; GC, germinal centre; LN, lymph node; CR, complete response; PR, partial response; PD, progressive disease; NA, not available; FCM, flow cytometry; IHC, immunohistochemistry.
